# Breeding for delayed bolting decelerated the circadian clock in cultivated lettuce

**DOI:** 10.1111/nph.70489

**Published:** 2025-08-21

**Authors:** Cèlia Anton‐Sales, Esther S. van den Bergh, Alejandro Thérèse‐Navarro, Edouard Severing, Daniel Moñino‐López, Joseph DiPalma, Marcel Proveniers, C. Robertson McClung, Marieke Jeuken, Guusje Bonnema

**Affiliations:** ^1^ Plant Breeding Wageningen University and Research (WUR) 6708 PB Wageningen the Netherlands; ^2^ Translational Plant Biology, Department of Biology Utrecht University 3584 CH Utrecht the Netherlands; ^3^ Department of Computer Science Dartmouth College Hanover NH 03755 USA; ^4^ Department of Biological Sciences Dartmouth College Hanover NH 03755 USA

**Keywords:** bolting, breeding, circadian clock, domestication, flowering time, genome‐wide association study (GWAS), lettuce (*Lactuca sativa*), PHYC

## Abstract

- Circadian clocks pace biological events and influence developmental traits, but their role in leafy crop domestication has remained unexplored. We investigated whether selection for delayed bolting during lettuce domestication targeted clock components.
- We phenotyped circadian rhythms and developmental timing across 234 cultivated and wild lettuce accessions. Using high‐throughput leaf movement tracking, genome‐wide association studies (GWAS) and haplotype analyses, we identified genetic variants controlling both the clock period and bolting time.
- Cultivated lettuce exhibits a significantly longer circadian period than its wild relatives, associated with a truncating mutation in *PHYTOCHROME C (PHYC).* This allele is not only associated with a decelerated clock but also with delayed bolting and flowering time in multiple field experiments. The truncating *PHYC* allele (H02) is enriched in modern cultivars and phylogenetically close to the wild ancestor (*Lactuca serriola*) alleles, indicating an early selection during lettuce domestication.
- Our study directly links for the first time circadian clock deceleration to domestication and breeding in a leafy crop. *PHYC* emerges as a pleiotropic regulator of the clock and developmental timing shaped by selecting delayed bolting during lettuce domestication and breeding. We demonstrate that circadian phenotyping is a powerful, scalable tool to predict developmental timing and uncover targets for crop improvement.

## Introduction

The circadian clock is a widespread biological timing system that enables organisms to anticipate and respond to daily environmental changes (Harmer, 2009). In plants, this endogenous oscillator governs numerous physiological and developmental processes (McClung, 2006), including photosynthesis, starch metabolism, growth and the transition to flowering. The clock consists of interlocked transcriptional feedback loops that generate *c*. 24‐h rhythms in gene expression.

Key core clock components include the morning‐phased transcription ***factors** CIRCADIAN CLOCK ASSOCIATED 1* (*CCA1*) and *LATE ELONGATED HYPOCOTYL* (*LHY*), which repress the evening‐expressed genes *EARLY FLOWERING 3* (*ELF3*), *EARLY FLOWERING 4* (*ELF4*) and *LUX ARRHYTHMO* (*LUX*), which in turn suppress morning gene expression. Additional pseudo‐response regulators (*PRR9*, *PRR7*, *PRR5* and *PRR1/TOC1*) modulate clock amplitude and fine‐tune transcriptional dynamics throughout the day (Sanchez *et al*., 2020). Together, these components form a complex and interconnected network that maintains circadian rhythmicity under constant conditions but also synchronizes it with external cues such as light and temperature through a process known as entrainment (Wang *et al*., 2022b).

Two fundamental properties characterize circadian rhythms: the free‐running period (τ, the intrinsic length of the cycle in the absence of cues) and the phase (φ, the timing of rhythmic events relative to the external environment). These parameters determine how biological activities align with daily cycles and influence plant performance. Alterations in clock parameters can have profound effects on growth and reproduction, particularly under seasonal or latitudinal variation (Greenham *et al*., 2017).

Crop domestication has repeatedly targeted circadian components, often unintentionally, to enhance adaptation to novel environments. In tomato, mutations in light‐signalling genes *EID1* and *LNK2* have been linked to clock deceleration and adaptation to higher latitudes (Müller *et al*., 2016, 2018). In cereal crops like wheat and barley, loss‐of‐function alleles in *PHYC* delay flowering (Nishida *et al*., 2013; Chen *et al*., 2014) while mutations in *ELF3* shorten it (Faure *et al*., 2012). Similarly, in soybean, a truncating allele of *GI* correlates with early flowering time (Wang *et al*., 2016). These modifications contribute to improved yield in different cultivation habitats and highlight that clock and clock‐related genes are frequent targets during domestication and breeding (McClung, 2021).

Lettuce (*Lactuca sativa*), a globally cultivated leafy vegetable, has undergone strong selection for delayed bolting, a trait that extends the harvest window, increases yield and improves head quality (Han *et al*., 2021). Early transition to flowering, or bolting, leads to stem elongation and reduced marketability due to bitterness and texture changes (Sessa *et al*., 2000; Assefa *et al*., 2019). Despite the economic importance of bolting time, the molecular basis of this trait has only been elucidated recently (Park *et al*., 2021; Wei *et al*., 2021; Wang *et al*., 2022a; Chen *et al*., 2025) and its potential connection to circadian function has not been previously explored.

In this study, we investigate natural variation in circadian rhythms across a very diverse panel of cultivated and wild lettuce accessions. Using high‐throughput leaf movement tracking, we quantify the circadian period and phase in 234 accessions. We integrate this data with genome‐wide association studies (GWAS) for developmental timing traits and identify *PHYTOCHROME C* (*PHYC*) as a shared regulator of clock function and bolting time. We demonstrate that a loss‐of‐function *PHYC* allele, prevalent in cultivated lettuce, causes circadian deceleration and delays reproductive development. Our findings provide evidence that the circadian clock has been modified through selection for delayed bolting, budding and flowering time and highlight *PHYC* as a key pleiotropic regulator in lettuce domestication.

## Materials and Methods

### Plant material

We analysed 445 *Lactuca* single‐seed descent lines from the Centre for Genetic Resources, the Netherlands (CGN; http://www.wur.eu/cgnsc002), previously described in detail (Wei *et al*., 2021). If not stated otherwise, our study set included 140 *Lactuca sativa*, 206 *Lactuca serriola* and 60 *Lactuca saligna* accessions, covering a wide range of geographic origins and cultivation types (Supporting Information Table S1). Where available, cultivation habits were curated from the International Monograph of Lettuce Varieties (Rodenburg *et al*., 1960).

### Plant growth conditions

Seeds underwent cold dark stratification (*c*. 4°C, 3 d) before germination. For circadian phenotyping, seedlings were grown on saturated filter paper under 12 h : 12 h, light : dark (LD) cycles (20°C : 18°C, *c*. 100 μmol m^−2^ s^−1^ white light) for 3 d; then transferred to soil‐filled pots and placed in a controlled chamber under constant light and temperature (LL, *c*. 100 μmol m^−2^ s^−1^, 20°C) for imaging.

For developmental phenotyping, 197 *L. sativa* and 200 *L. serriola* accessions were field‐grown in Maasbree, the Netherlands. Two replicate plots (30–40 plants each) per accession were sown in March 2021 and transplanted in April (Fig. S1) and phenotyped both destructively and nondestructively (Dijkhuizen *et al*., 2025). Bolting and reproductive stages were scored biweekly using the BBCH scale (Feller *et al*., 1995) (Table 1; Fig. S2).

**Table 1:** Descriptions of the BBCH‐scale (0–9) reproductive transitions (5–6) for leafy vegetables.

Growth stage	Code	Description
5. Inflorescence emergence	51	The main shoot (inside the head) begins to elongate
	55	First individual flowers of main inflorescence visible (still closed)
6. Flowering	60	First flowers open (sporadically)

For head‐forming leafy vegetables, the main shoot begins to elongate inside the head when bolting begins (51), then inflorescence organs develop (55) and flowers open (60).

Destructive measurements were performed weekly from June to mid‐July, assessing five plants per plot (reduced to three from Day 89 onward). Phenotyping data for flowering time of 132 publicly resequenced *L. sativa* accessions (Wei *et al*., 2021) was also compiled.

### Circadian period and phase estimation

Leaf movement was monitored as described in detail before (Greenham *et al*., 2015; Lou *et al*., 2022). In short, we used nine Canon PowerShot ELPH 300 HS cameras (CHDK‐configured) imaging *c*. 60 cotyledons per frame every 20 min for 7 d under constant conditions. A randomized block design (three replicates per genotype, 20 genotypes per tray) was generated using FielDHub (Florez, 2024).

Leaf position was quantified using the TRiP Matlab pipeline (Greenham *et al*., 2015). Period and phase were estimated via FFT‐based analysis with Nelder–Mead optimization. We applied RAE filtering (< 0.19), removed out‐of‐range values (τ < 21 or > 30 h) and retained accessions with ≥ 3 replicates, yielding reliable data for 236 genotypes (Table S2).

### Variant calling with v11 lettuce genome

Publicly available raw sequencing reads (Wei *et al*., 2021) were trimmed using Trimmomatic (v.0.39) (Bolger *et al*., 2014), aligned to the *L. sativa* cv Salinas latest genome (v11, GCF_002870075.4) using Bwa‐Mem (v.0.7.17) (Vasimuddin *et al*., 2019) and processed with samtools (v.1.14) (Li *et al*., 2009) to mark duplicates, sort and index. Variants were called with bcftools using mpileup and call, and filtered for quality (DP > 4, QUAL > 20), normalized and merged across samples. The resulting VCF contained *c*. 485 M SNPs across 450 accessions.

### Genome‐wide association studies (GWAS)

GWAS was performed using the Efficient Mixed‐Model Association eXpedited (EMMAX) algorithm, as described before (Kang *et al*., 2010) on multiple developmental stages of the lettuce growth and life cycle as well as on circadian clock periodicity (see Table S3). The phenotypic data for developmental stages included: Bolting Time (Days until 51, Utrecht University (UU), 2021), Flowering Time (CGN, 2021, and Days until 60, UU, 2021), Inflorescence Occurrence (Days until 55, UU, 2021), Destructive Start Bolting (Days until stage 1, UU, 2021), Destructive Start Budding (Days until stage 2, UU, 2021), Destructive Bolting (Days until stage 3, UU, 2021) and Destructive Budding (Days until stage 4, UU, 2021). Thus, we had two independent datasets for flowering time (CGN and UU), two for bolting time (destructive and nondestructive), two intermediate stages (start bolting destructive, start budding destructive), inflorescence emergence and circadian clock, accounting for a total of nine independent GWAS.

SNPs were filtered for quality (QUAL > 10, MQ > 30), biallelic status, MAF ≥ 0.05 and missingness ≤ 0.1. A dynamic filtering pipeline was applied using bcftools on read depth and bias metrics (MQBZ, RPBZ, SCBZ) and LD pruning (*r* ^2^ > 0.8 in 10 kb windows) was performed using PLINK (Purcell *et al*., 2007). The final SNP set included 2186 682 high‐confidence variants across 132 *L. sativa* accessions (Fig. S3).

Population structure was corrected using PCA (top 5 PCs as covariates, Fig. S4) and a kinship matrix (Balding‐Nichols), both included in the EMMAX model. Bonferroni correction was applied (α = 0.05, *P* < 2.29 × 10^−8^).

To assess the effect of the lead *PHYC* SNP (Chr7:176812430), we re‐ran GWAS with EMMAX; including this SNP as a covariate (known effect).

We additionally tested GWAS using the rmvp package (Yin *et al*., 2021) with additional GLM, MLM and FarmCPU models, incorporating three to five PCs and kinship. All GWAS outputs were visualized and clumped using GWASLab (He *et al*., 2023).

### PHYC haplotype phasing

PHYC haplotypes were phased from the filtered VCF using Beagle (v.5.434) (Browning *et al*., 2021) (10 burn‐in, 50 phasing iterations). Variants were grouped into haplotypes if they differed by ≤ 1.4% (≤ 7 mismatches among 492 SNPs). A custom pipeline for haplotype clustering is available at https://github.com/alethere/hapgroup.

### Haplotype strength prediction

We assessed the predictive power of *PHYC* haplotypes present in cultivated lettuce (H02 vs H03) on key traits using a receiver operating characteristic (ROC) curve analysis (DeLong *et al*., 1988; Kamitsuji & Kamatani, 2006; Pers *et al*., 2015). The phenotypic traits were used as continuous predictors and haplotypes coded as a binary variable. The area under the curve (AUC) values were calculated via the Mann–Whitney *U* statistic to measure discrimination between haplotypes (DeLong *et al*., 1988). Cohen's *d* and Mann–Whitney tests assessed effect size and significance. Bootstrap resampling provided 95% confidence intervals for AUC estimates, quantifying haplotype–phenotype associations for circadian period, bolting and flowering time.

### Phylogenetic and protein domain analysis

Pairwise distances between phased haplotypes were calculated using MEGA (Tamura *et al*., 2021) and used to construct a neighbour‐joining tree (Ape v.5.0) visualized with ggtree (Yu *et al*., 2017). Protein‐coding sequences of each PHYC haplotype were reconstructed and translated using Biopython (v.1.84) (Cock *et al*., 2009), aligned with Mega and annotated for domain structure using conserved motif prediction.

## Results

### Cultivated lettuce has a decelerated circadian clock

To assess circadian variation, we monitored rhythmic leaf movement under constant light and temperature in 234 *Lactuca* accessions. The panel included 134 *L. sativa* cultivars, 56 *L. serriola* (wild ancestor) and 44 *L. saligna* accessions (wild relative). Leaf position was imaged every 20 min over 7 d, and circadian parameters were estimated using FFT‐based analysis and iterative Nelder–Mead optimization. Period and phase estimates were filtered for rhythm robustness and replicate consistency.

**Fig. 1:**
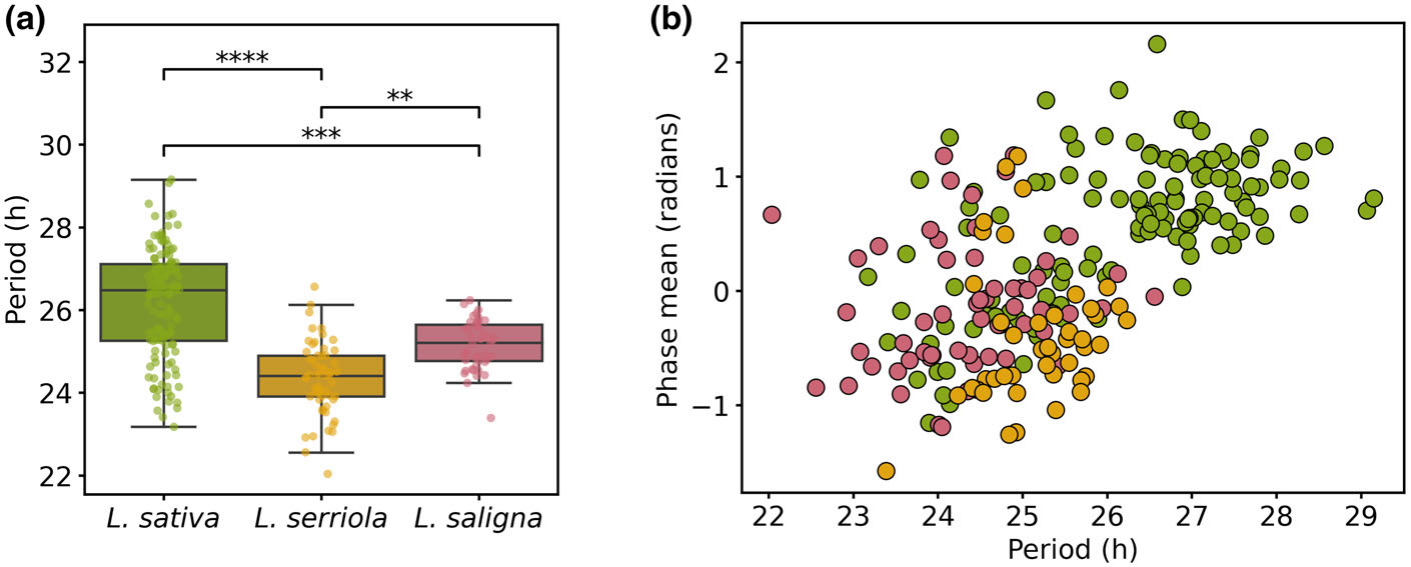
Domestication slowed down the circadian clock in lettuce. (a) Boxplot illustrating the circadian clock period (in hours) for three species: *Lactuca sativa* (green), *Lactuca serriola* (yellow) and *Lactuca saligna* (pink). Boxplots show median (central line), interquartile range (box), whiskers extending to data range. Each dot represents the mean period for at least three replicates of the same genotype (*n* ≥ 3). Statistical significance is indicated by asterisks (****, *P* < 0.0001; ***, *P* < 0.001; **, *P* < 0.01, Mann–Whitney *U*). (b) Scatterplot showing the relationship between circadian period and phase angle (in radians) for each species (color‐coded as in a.). Each dot represents the average period and phase angle for a single accession (*n* ≥ 3).

*Lactuca sativa* exhibited a significantly longer circadian period (mean = 26.17 h) compared to its direct ancestor *L. serriola* (24.34 h) and the distantly related *L. saligna* (25.15 h) (Fig. 1a). Most *L. sativa* accessions had periods between 26 and 28 h, while wild species periods clustered closer to 24 h. This represents a clear deceleration of the circadian clock in domesticated lettuce. The phase angle of leaf movement was positively correlated with period length (Spearman *r* = 0.59, *P* < 1· 10–20), indicating a consistent delay in peak biological timing with longer periods (Figs 1b, S5a).

Notably, the variation in period length among *L. sativa* accessions was not associated with geographic origin but showed enrichment by cultivar type. Accessions with near‐24 h rhythms were predominantly winter cultivars, landraces, stalk and oilseed types. Those with extended periods of 27 h included modern leaf‐type cultivars selected for long‐day cultivation (Table S4a,b). This suggests that clock deceleration might reflect targeted selection for delayed bolting in long‐day bolting‐inducing environments.

We quantified rhythm robustness using relative amplitude error (RAE), a measure where 0 represents an ideal cosine wave and 1 marks the threshold of statistical significance. Cultivated lettuce showed significantly lower RAE values than wild species (Fig. S5b), indicating more stable circadian rhythms under constant conditions despite greater period variation among cultivated accessions.

While larger cotyledons in cultivated lettuce may contribute to more stable leave movement readings, this pattern could also suggest that domestication has selected for circadian clocks capable of maintaining highly robust self‐sustained rhythms.

### GWAS reveals a shared genetic basis for clock and developmental timing

To uncover genetic loci underlying circadian and developmental variation, we performed GWAS on 132 resequenced *L. sativa* accessions mapped to the latest lettuce genome (cv Salinas, v11 GCF_002870075.4). Phenotypes included the circadian period and multiple developmental traits scored in field conditions: bolting time, budding, inflorescence emergence and flowering time. Destructive and nondestructive measurements were taken and used for GWAS on developmental traits; publicly available flowering time data was used for comparison.

**Fig. 2:**
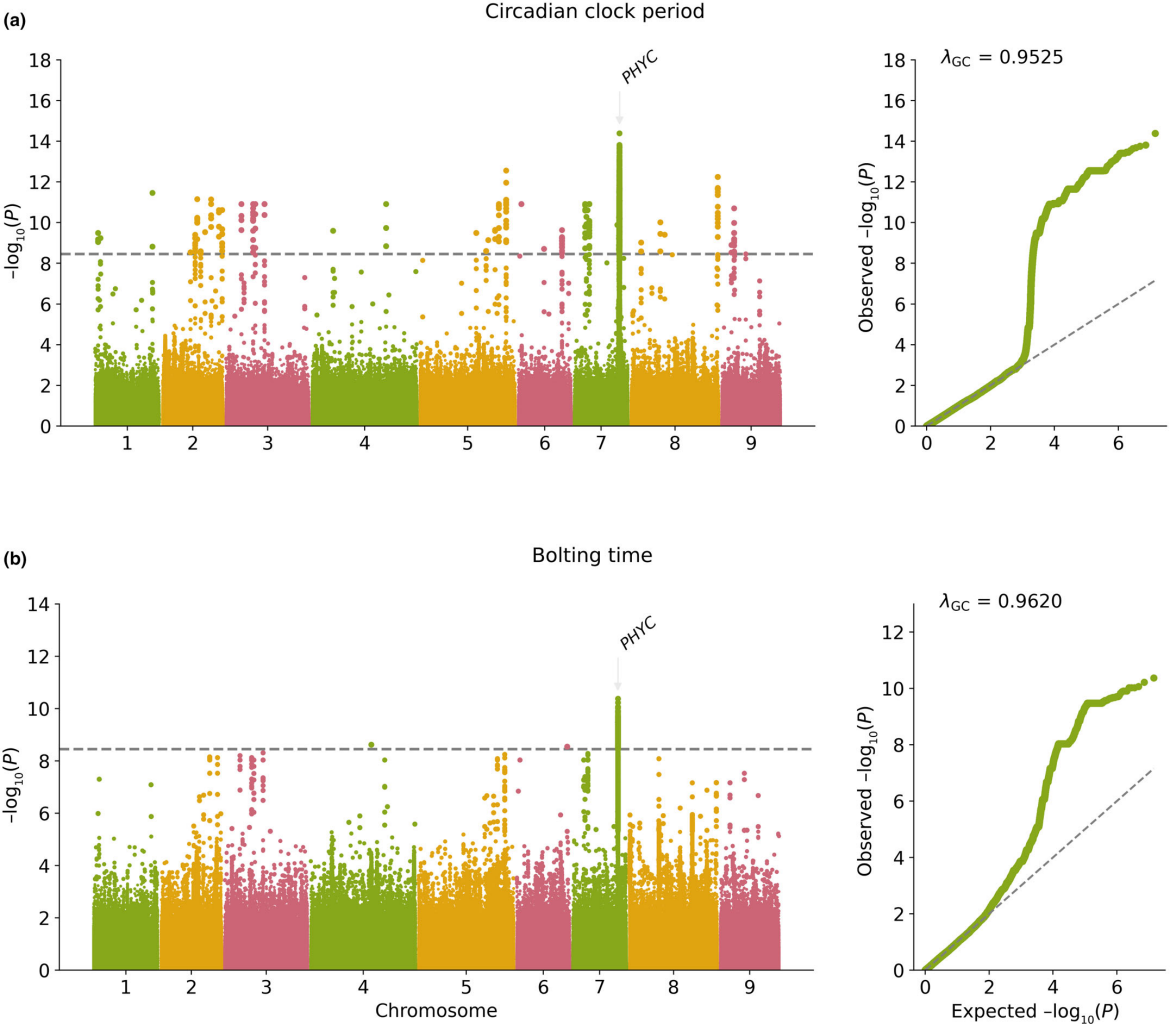
Genome‐wide association analysis (GWAS) of circadian clock periodicity and bolting time in *Lactuca sativa*. (a) Manhattan plot and quantile–quantile plot (*Q*–*Q* plot) showing the GWAS results for circadian clock periodicity across the cultivated lettuce genome. The dashed horizontal grey line represents the Bonferroni‐corrected significance threshold (α = 0.05, 2.29 · 10^−8^). A significant association is observed on Chr. 7: 166–168 Mb, with our candidate gene *PHYTOCHROME C* (*PHYC*), indicated. This locus exhibits the strongest association with variation in circadian periodicity among genotypes. (b) Manhattan and *Q*–*Q* plots illustrating GWAS results for bolting time (days to 51 in BBCH scale). The dashed horizontal grey line marks the Bonferroni‐corrected significance threshold (α = 0.05, 2.29 · 10^−8^). The same peak on chromosome 7 highlights *PHYC* as the most significant locus associated with bolting time. The genomic inflation factor (λGC) is indicated in each *Q*–*Q* plot.

Strikingly, we detected a strong association signal on chromosome 7 (176.6–176.8 Mb) that was found across all GWAS on developmental traits as well as for circadian period (Figs 2, S6). The lead SNP on this peak was located at Chr7:176812430 (Fig. S7; Table S5). This genomic region contains the gene *PHTOCHROME C* (*PHYC*) (Chr7:176809301–176814445), a red/far‐red light photoreceptor with known roles in flowering and light signalling (Chen *et al*., 2025) (Table S6).

The association was robust across all the GWAS models tested (EMMAX, GLM, MLM and FarmCPU) (Fig. S8; Table S7), and the lead SNP at Chr7:176812430 was consistently found with the lowest *P*‐value in all our GWAS.

To test whether this represents a single causal locus, we performed additional analysis by including the lead SNP (Chr7:176812430) as a covariate in our GWAS models. When doing so, all significant association signals were lost, confirming that variation at this single major‐effect locus drives the observed phenotypic associations across all developmental traits and circadian period (Fig. S9).

Thus, *PHYC* consistently emerged as the primary candidate gene linking circadian period and developmental timing in lettuce. In Arabidopsis and cereals (Balasubramanian *et al*., 2006; Chen *et al*., 2014) *PHYC* participates in photoperiodic flowering through light perception and interaction with circadian components. However, its role in directly modulating the circadian period itself has not been previously demonstrated in leafy crops; its functions connecting light sensing, clock function and developmental transitions in vegetable species are proposed and described here for the first time.

### A truncating *PHYC* allele was selected during lettuce domestication

**Fig. 3:**
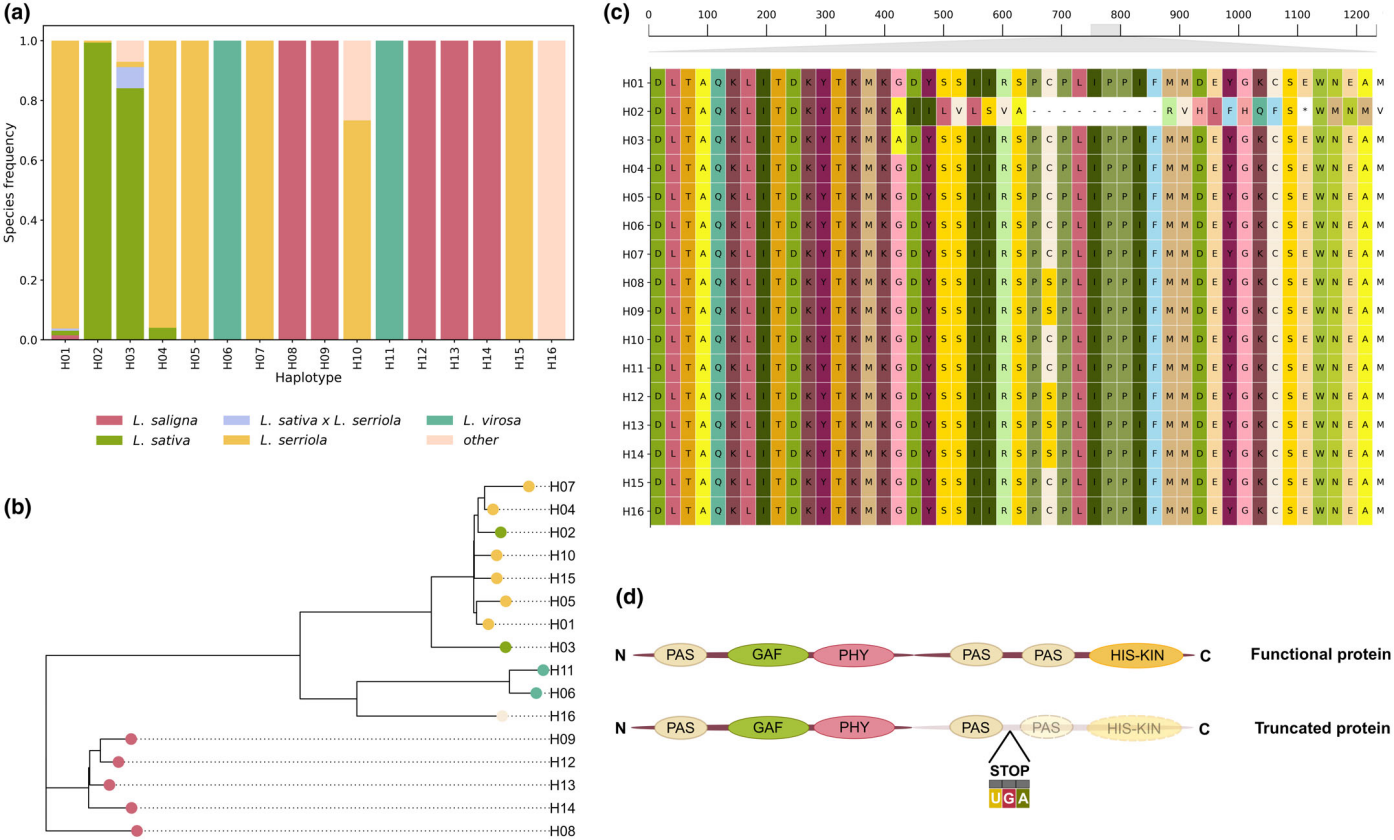
Analysis of *PHYC* haplotypes in our *Lactuca* collection, their phylogeny and protein alignment. (a) Stacked bar plot illustrating the distribution of species frequencies across *PHYC* haplotypes (H01–H16), with a minimum count threshold of five. Each bar represents a haplotype, with colours indicating the relative proportions of species as occurrence frequencies within each species. (b) Phylogenetic tree showing evolutionary relationships between representative variants of *PHYC* haplotypes in the *Lactuca* genus (H01–H16). Each tip represents a reference variant for a specific haplotype, with species assignment based on the most frequently observed species carrying that haplotype (as indicated by colour coding from panel a). Branch lengths are proportional to genetic distance between variants, calculated from sequence similarity data, where longer branches indicate greater evolutionary divergence. (c) Multiple sequence alignment of PHYC protein variants (H01–H16), highlighting key conserved and variable regions. Rows represent haplotypes, and columns correspond to amino acid positions. Residues are color‐coded. The deletion specific to H02 (GA → G‐) leading to a frameshift mutation and truncation of the PHYC protein causes the gap in the alignment leading to an early stop codon. Gaps are indicated by dashes (−), and the stop codon is represented by an asterisk (*). The scale shows the zoomed‐in alignment spanning positions 750–800 of the total 1234 amino acids in the full‐length protein. (d) Schematic representation of the PHYC protein structure for functional and truncated variants. The functional protein (top) includes all predicted conserved domains (PAS, GAF, PHY and HIS‐KIN) essential for light perception, stabilization, dimerization and nuclear localization of phytochromes. The truncated protein (bottom), as observed in haplotype H02, lacks the PAS and HIS‐KIN domains, which likely impair its nuclear function. The site of the frameshift mutation introducing the early stop codon (UGA) is indicated.

To further examine the effects of our lead SNP within the *PHYC* locus, we phased *PHYC* haplotypes across our panel of 445 *Lactuca* accessions. We identified 16 distinct *PHYC* haplotypes (Fig. 3a), with three major forms (Fig. S10; Table S8): H01 (full‐length), H02 (truncating) and H03 (full‐length).

H01 is present in 97 accessions (primarily *L. serriola*) and, when translated, encodes a full‐length PHYC protein of 1234 amino acids, with all the functional domains described for phytochrome proteins (Fig. 3c,d) (Nagatani, 2005; Burgie & Vierstra, 2014).

By contrast, H02 is found in 89 accessions (88 *L. sativa* and 1 hybrid), and harbours a GA → G deletion in exon 2 that induces a frameshift and a premature stop codon (Fig. 3c). This mutation results in a truncated 768‐amino acid protein. Specifically, the H02 protein lacks the C‐terminal PAS and HIS‐KIN domains essential for signal transduction and nuclear localization (Fig. 3d). Notably, H02 represents the reference allele in the *Lactuca sativa* genome assembly (v11), and the lead SNP identified across all GWAS analyses (Chr7:176812430) corresponds precisely to this GA → G deletion.

Phylogenetic analyses (Fig. 3b) show that the truncating H02 clusters closely with the wild‐type *L. serriola* clade, indicating recent introgression with minimal postintrogression divergence. This suggests that H02 originated from wild germplasm and was subsequently introgressed into cultivated lettuce early on during lettuce domestication.

The third major haplotype, H03, is found in 58 accessions. This haplotype encodes a full‐length and a predictably functional PHYC protein, found in 48 accessions of cultivated lettuce, 4 from *sativa* × *serriola* hybrids and 5 from other groups. Within cultivated lettuce, H03 is more commonly found in winter or less‐bred cultivars, landraces and speciality morphotypes such as celtuce or oilseed types (Table S8), suggesting it may harbour adaptive variants selected for specific agricultural niches or environmental conditions. Concretely, H03 forms a divergent and isolated phylogenetic clade (Fig. 3b), consistent with an independent domestication event or ancient breeding lineage maintained separately from the main cultivated lettuce phylogenetic trajectory.

### Haplotype–phenotype analysis reveals coordinated clock‐development control

To elucidate the effect of the truncating H02, we conducted haplotype–phenotype associations for the three major haplotypes found in our collection (H01, H02 and H03); further, we compared the two unique haplotypes found in cultivated lettuce (H02 and H03).

**Fig. 4:**
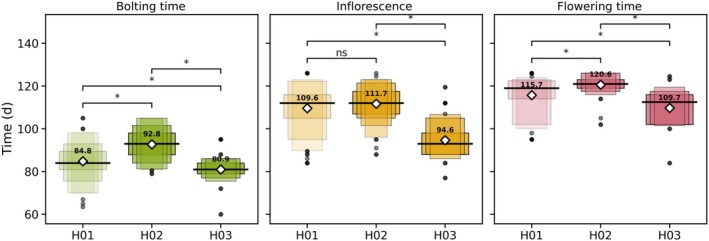
Time to bolting (green), inflorescence emergence (yellow) and flowering (rose) across the three major *PHYC* haplotypes found in our lettuce collection. H01, found exclusively in *Lactuca serriola*, the wild ancestor of cultivated lettuce, is displayed with reduced colour intensity to reflect its absence in cultivated lettuce. H02 is a truncated haplotype common in *Lactuca sativa*, while H03 is a functional allele present in fewer cultivated accessions. Each plot displays nested boxes representing successive letter values (quantiles), where the outermost box shows the interquartile range (25^th^–75^th^ percentiles) and inner boxes represent progressively more extreme quantiles. Diamonds indicate group means and thick black lines show medians. Pairwise statistical comparisons are shown with significance levels (*P*‐value < 0.05: *, Mann–Whitney *U*; ns, not significant). Phenotypic data correspond to time to bolting (days to 51), inflorescence emergence (time to 55) and flowering time (days to 60).

The H01 haplotype, prevalent in *L. serriola*, encodes a full‐length PHYC protein predicted to be fully functional and exhibits an early onset of reproductive development, with bolting time of 85 d. H03, found in a subset of cultivated lettuce accessions, also encodes a functional PHYC protein, and accessions carrying this allele bolt on average after 81 d. By contrast, accessions carrying the H02 truncating allele exhibit significantly delayed bolting times (93 d on average) compared to those with the functional alleles H01 and H03 (Fig. 4). Further differences in reproductive timing were also found. On average, H02 delays bolting by 8–12 d, inflorescence emergence by 2–17 d and flowering by 5–11 d. These effects were consistent across destructive and nondestructive measurements and flowering time data from two independent field experiments.

**Fig. 5:**
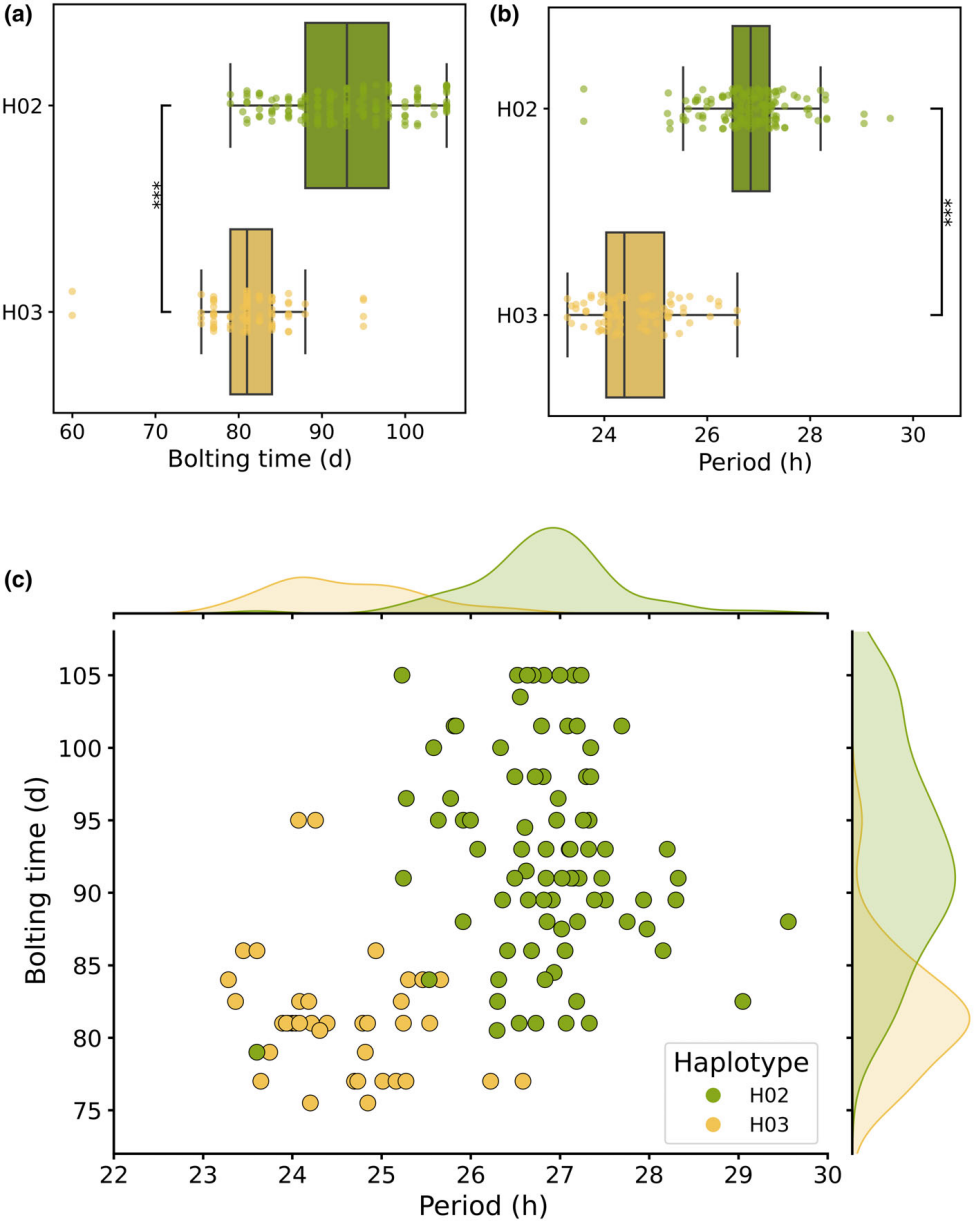
Association of the H02 haplotype of *PHYC* with delayed bolting, extended circadian period and the distribution of H02 and H03 across cultivated lettuce. (a, b) Boxplots showing bolting time and period for the two major cultivated lettuce haplotypes, H02 (olive) and H03 (gold). The H02 haplotype is associated with significantly delayed bolting and a decelerated circadian clock. Statistical significance was tested using the Mann–Whitney *U* test (***, *P* < 0.0001). Boxplots show median (central line), interquartile range (box) and whiskers extending to data range. Each dot represents the mean period for at least three replicates of the same genotype (*n* ≥ 3). (c) Scatterplot illustrating the correlation between bolting time (*y*‐axis) and circadian period length (*x*‐axis) for H02 and H03 haplotypes. Spearman correlation (ρ = 0.488, *P* = 7.7 × 10^−9^). Points represent individual accession means (*n* ≥ 3), with the H02 haplotype clustering toward longer periods and delayed bolting times, while H03 exhibits shorter periods and earlier bolting. The marginal histograms show on the top the distribution of circadian periods for each haplotype (H02 olive, H03 gold) and on the right side, the distribution of bolting types for each haplotype (H02 olive, H03 gold).

Haplotype–phenotype association analyses comparing the two major haplotypes found in cultivated lettuce (H02 and H03) confirmed that H02 not only alters developmental timing but also significantly decelerates the circadian clock (Fig. 5). Cultivated lettuce accessions carrying H02 exhibit longer circadian periods (mean = 26.8 h) than those carrying H03 (mean = 24.6 h), paired with significantly delayed bolting times (93 vs 81 d) (Fig. 5a,b). This association is visualized in the period‐bolting time scatterplot (Fig. 5c), where H02 accessions cluster toward longer periods and later bolting, while H03 accessions show shorter periods and earlier bolting, supported by a moderately positive Spearman correlation (ρ = 0.488, *P* = 7.7 × 10^−9^).

Together, these results support the hypothesis that the truncating H02 might disrupt PHYC light‐signalling function, thereby decelerating the circadian oscillator and delaying developmental transitions.

### Quantitative assessment confirms binary phenotypic effects of H02

To quantify the strength of our haplotype–phenotype associations and assess the H02's effects, we performed receiver operating characteristic (ROC) analysis across 132 *L. sativa* accessions for which we had phenotypic data (85 H02 and 47 H03). This analysis treats H02 presence as a binary predictor for a certain trait and evaluates how accurately it discriminates between contrasting phenotypes. Specifically, we tested whether H02 presence predicts decelerated circadian clocks and delayed developmental timing, while H02 absence (and thus, H03 presence) predicts *c*. 24‐h clocks and earlier bolting and flowering times.

The ROC analysis revealed exceptional discriminative power for the H02 haplotype across the measured traits (Table 2). For circadian period prediction, the AUC reached 0.968 (95% CI: 0.936–0.993) (Fig. S11), indicating that H02 presence predicts circadian deceleration with *c*. 97% accuracy. Similarly, exceptional predictive performance was observed for developmental traits, with AUCs of 0.910 (95% CI: 0.856–0.957) for bolting time and 0.911 (95% CI: 0.855–0.954) for flowering time prediction. Notably, the effect sizes for all traits were very large (Cohen's *d* = 1.77–2.69), well above the 0.8 threshold for large effects, indicating that haplotype differences represent substantial biological changes.

**Table 2:** Phenotypic effects and discriminative power of *PHYC* haplotypes in cultivated lettuce.

Trait	H02 Mean	H03 Mean	Difference	Effect size (Cohen's *d*)	AUC	95% CI	*P*‐value
Circadian period (h)	26.8	24.6	2.2	2.69	0.968	0.936–0.993	2.04 × 10^−17^
Bolting time (d)	92.7	81.2	11.5	1.77	0.91	0.856–0.957	8.44 × 10^−15^
Flowering time (d)	123.7	104.2	19.5	1.79	0.911	0.855–0.954	1.62 × 10^−15^

Quantitative comparison of bolting (days until 51), flowering time (CGN) and circadian traits between H02 and H03 haplotypes. Receiver operating characteristic (ROC) analysis was performed on 132 *Lactuca sativa* accessions (85 H02, 47 H03). Area under the ROC curve (AUC) values > 0.9 indicate excellent discrimination. Effect sizes calculated using Cohen's *d* > 0.8 are considered large. Statistical significance (*P*‐value) assessed using Mann–Whitney *U* tests. CI, confidence interval. Cohen's *d* = (Mean_1_–Mean_2_)/Pooled Standard Deviation.

These results demonstrate that *PHYC* haplotype variation can serve as a highly accurate molecular marker for lettuce breeding programs. The highly strong predictive power of the H02 allele (91% accuracy) enables breeders to predict bolting time based on *PHYC* haplotype identity; facilitating marker‐assisted selection for delayed reproductive development in cultivated lettuce.

## Discussion

Our multi‐species‐wide study demonstrates that domesticated lettuce exhibits a decelerated circadian clock when compared to its wild relatives, which we have associated with a truncating mutation in *PHYTOCHROME C* (*PHYC*). This frame‐shift mutation is also linked to delayed bolting and flowering times, traits that have long been favoured during domestication and breeding to extend the vegetative growth phase and thereby enhance lettuce yield.

Interestingly, the impact of C‐terminal *PHYC* mutations – like the one reported in this study – appears to be species dependent. In wheat, mutations in the C‐terminal domain of *PHYC* lead to a complete loss of function, disrupting its essential roles in photoperiodic signalling and flowering time regulation (Chen *et al*., 2014). This highlights the critical role of *PHYC* in wheat. By contrast, studies in *Arabidopsis thaliana* indicate that similar C‐terminal mutations do not significantly impair PHYC function, implying a lesser role in signalling, likely compensated by other phytochromes such as PHYB and PHYA (Wang *et al*., 2023).

Given that cultivated lettuce exhibits a severe developmental phenotype when PHYC is truncated at the C‐terminus (from 1234 to 768 amino acids), it is likely that *PHYC* in lettuce plays a similarly crucial pleiotropic role in photoperiod sensing and circadian regulation as reported in grasses, serving as a key circadian promoter of bolting and flowering under long‐day conditions. Consistent with this hypothesis, recent studies in lettuce have identified *PHYC* associations with flowering and bolting time (Wei *et al*., 2021; Chen *et al*., 2025); however, the links between circadian modulation and reproductive time are reported in this study for the first time.

Our proposed model is supported by our haplotype–phenotype associations, where H02 accessions exhibit delayed circadian periods (mean = 26.8 h) coupled with delayed bolting and flowering times. Further, our ROC analysis provides compelling quantitative evidence for H02's role as a phenotypic determinant. With AUC values of 0.97 for circadian period and 0.91 for developmental traits, H02 presence predicts phenotypic outcomes with 91–97% accuracy, indicative of excellent discriminative power. These results demonstrate that *PHYC* variation functions as a highly effective molecular determinant of both circadian and developmental timing, with great potential as a molecular marker for breeding programs.

Integrating our phylogenetic analysis, protein structural predictions and phenotypic data reveals a compelling domestication narrative for delayed bolting in lettuce. The widespread distribution of H02 across *L. sativa* accessions, contrasted with its absence in wild species, suggests that the ancestral cultivated allele (H03) was progressively replaced by H02 through introgression from wild *L. serriola* populations. This pattern indicates that wild *L. serriola* contributed either a pre‐existing truncated *PHYC* allele or an ancestral variant that subsequently led to the truncated form found in lettuce cultivars. The predominance of H02 in cultivated lettuce varieties reflects the genetic bottleneck characteristic of crop domestication, where traits conferring agricultural advantages – in this case, delayed reproductive transitions – become fixed through intensive artificial selection. Accordingly, the wild‐type H03 from cultivated lettuce, clustered in a phylogenetically distant and unique clade.

Together, our findings parallel domestication events in tomato (Müller *et al*., 2016, 2018), potato (Kloosterman *et al*., 2013), grasses (Nishida *et al*., 2013; Chen *et al*., 2014; Gao *et al*., 2023) and soybean (Wang *et al*., 2016; Greenham *et al*., 2017) where altering circadian clock and clock‐related components supported adaptation to new environments and improved local performance. However, lettuce provides a distinct case: here, the clock was decelerated not to adapt the crop to new environments or to shift flowering for seed or fruit production, but to prolong leaf growth before reproductive development. This highlights how circadian modifications can support diverse breeding targets depending on the harvested organ.

Importantly, we show that the circadian phenotype can serve as a predictive trait in breeding. Our work clearly reveals that when coupled with GWAS, leaf movement phenotyping of the circadian clock can aid the discovery of candidate genes. Since leaf movement tracking is nondestructive, scalable and applicable in early development, it offers a powerful tool to gain early insight of clock‐controlled traits like bolting or flowering time without requiring full growth cycles in highly variable field conditions. This accelerates selection and enables circadian‐informed breeding strategies.

In summary, we associate the truncation of PHYC to a circadian deceleration and delayed bolting in cultivated lettuce, adding to the growing evidence that circadian systems are key, yet unintended targets in crop domestication and improvement. By uncovering a clock‐development connection in a leafy vegetable, we broaden the understanding of how biological timing can be of enormous value for agronomical traits.

## Competing interests

None declared.

## Author contributions

CA‐S: Conceptualization, Methodology, Formal analysis, Investigation, Writing Manuscript, Visualization, Review and Editing. ESB: Methodology, Review. AT‐N: Formal analysis, Methodology, Review. ES: Formal analysis, Methodology, Review. DM‐L: Methodology, Supervision. JDP: Methodology. MP: Supervision. CRM: Conceptualization, Supervision, Facilities Assistance, Review. MJ: Conceptualization, Investigation, Review, Supervision. GB: Conceptualization, Investigation, Review, Supervision, Project Administration, Funding Acquisition.

## Disclaimer

The New Phytologist Foundation remains neutral with regard to jurisdictional claims in maps and in any institutional affiliations.

## Supporting information


**Fig. S1** Drone‐captured top view of the experimental field used for scoring developmental traits.
**Fig. S2** Chronological representation of four bolting stages scored through destructive measurements.
**Fig. S3** Distribution of SNPs after hard filtering and LD pruning.
**Fig. S4** SNP‐based PCA plot of 132 *Lactuca sativa* accessions used for the primary GWAS showing the first (30.5% variance explained) and second (15.2% variance explained) principal components (PCs).
**Fig. S5** Further examination of the cultivated lettuce extended circadian period.
**Fig. S6** Genome‐wide Association Analysis (GWAS) of developmental traits in *Lactuca sativa* (genome v.11).
**Fig. S7** Regional Manhattan plot of GWAS results for circadian clock period.
**Fig. S8** Genome‐wide association analysis (GWAS) of developmental traits and circadian clock in *Lactuca sativa* (genome v.11).
**Fig. S9** Genome‐wide association analysis (GWAS) of circadian clock and developmental traits in *Lactuca sativa* (genome v.11) with key SNP in the *PHYC* locus (Chr7:176812430) as known effect.
**Fig. S10** Validation of *PHYC* variant distribution across Lactuca species and haplotype assignments.
**Fig. S11** Receiver operating characteristic (ROC) analysis illustrating the discriminative power of the *PHYC* lettuce H02 haplotype.

**Table S1** Accessions used in this study with publicly available data from the CGN.
**Table S2** Circadian clock data of 236 accessions.
**Table S3** Developmental traits and circadian clock period of 132 *Lactuca sativa* accessions used for genome‐wide association analyses.
**Table S4** Circadian clock period, bolting time, inflorescence emergence and flowering time by culture type (2a) and crop type (2b).
**Table S5** Identified lead variants from the GWAS results.
**Table S6** Candidate genes associated with developmental traits and circadian clock.
**Table S7** Significant variants from the GWAS results using three different algorithms for confirmation.
**Table S8** Phased PHYC lettuce haplotypes in our collection (*n* = 445).
Please note: Wiley is not responsible for the content or functionality of any Supporting Information supplied by the authors. Any queries (other than missing material) should be directed to the *New Phytologist* Central Office.

## Data Availability

Sequence data from this article can be found in the Sequence Read Archive (under BioProject accession PRJNA693894, https://www.ncbi.nlm.nih.gov/bioproject/693894) and CNGB Nucleotide Sequence Archive (CNSA; under the accession number CNP0000335, https://db.cngb.org/data_resources/project/CNP0000335) (Wei *et al*., 2021).
