# Supplementary material for: Breeding for delayed bolting decelerated the circadian clock in cultivated lettuce
Source: New Phytol. 2025 Aug 21;248(4):1892–902. doi: 10.1111/nph.70489 (PMC12529022; doi:10.1111/nph.70489)

New Phytologist **Supporting Information**

Article title: **Breeding for Delayed Bolting Decelerated the Circadian Clock in Cultivated Lettuce**

Authors: Cèlia Anton-Sales, Esther S. van den Bergh, Alejandro Thérèse-Navarro, Edouard Severing, Daniel Moñino-López, Joseph Di Palma, Marcel Proveniers, C. Robertson McClung, Marieke Jeuken, Guusje Bonnema.

Article acceptance date: 30 July 2025

The following Supporting Information is available for this article:

Fig. S1 ***Drone-captured top-view of the experimental field used for scoring developmental traits.*** *Each block consists of 30-40 plants belonging to the same lettuce (Lactuca sativa) accession, with two blocks sown per accession. Blocks are visually separated by red lettuce for easier scoring and distinction. The arrangement of blocks follows an ascending TKI number order for the first block and a randomized complete block design (RCBD) for the second block.*

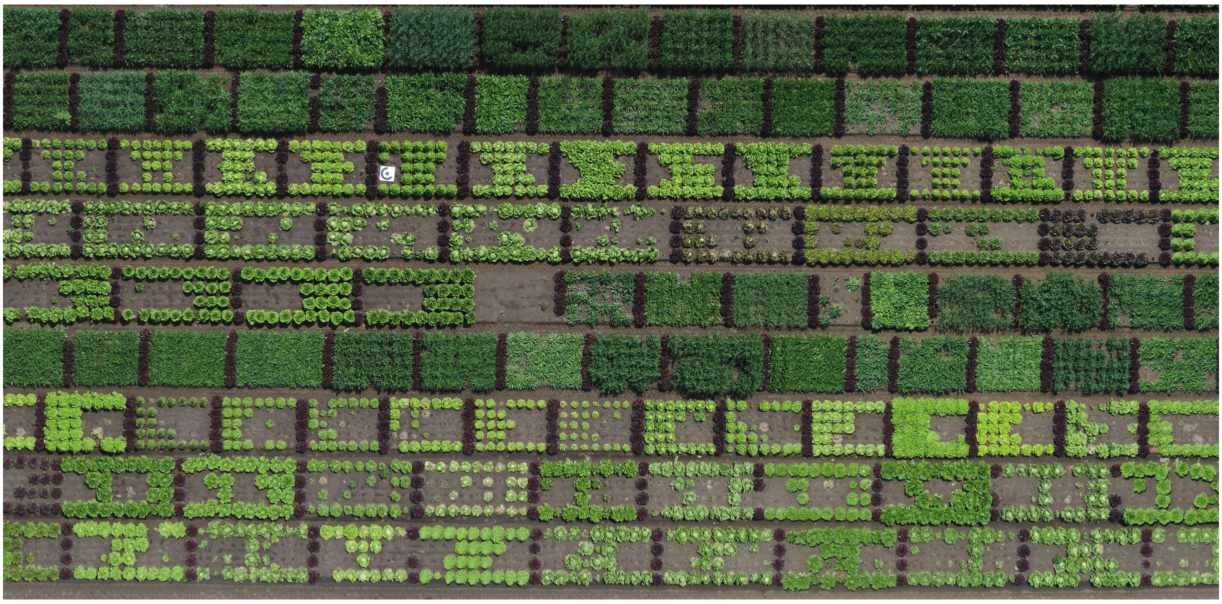

Fig. S2 ***Chronological representation of four lettuce (L. sativa) bolting stages scored through destructive measurements.*** *The stages depicted are from left to right: (1) start bolting, (2) bolting, (3) start budding, and (4) budding. An additional stage, 0 (no bolting), is not shown. Each stage illustrates the progression from vegetative growth to the reproductive phase.*

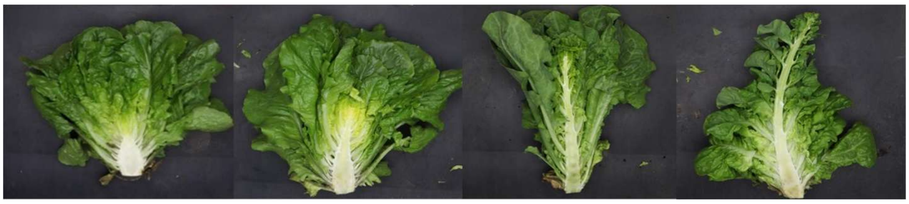

Fig. S3 ***Distribution of SNPs along the lettuce genome (v.11) after hard-filtering and LD pruning.*** *SNPs were filtered based on bcftools dynamic filtering guidelines, considering sequencing depth and quality scores, and subsequently LD-pruned to minimize redundancy among correlated SNPs. This pipeline resulted in a final dataset of 2,186,682 high-quality SNPs from an initial set of 484,854,932 evenly distributed across the lettuce genome. The color scale represents SNP density, ranging from dark blue (1 SNPs) to bright green (>6345 SNPs) within each 1Mb window. Gray indicates absence of SNPs.*

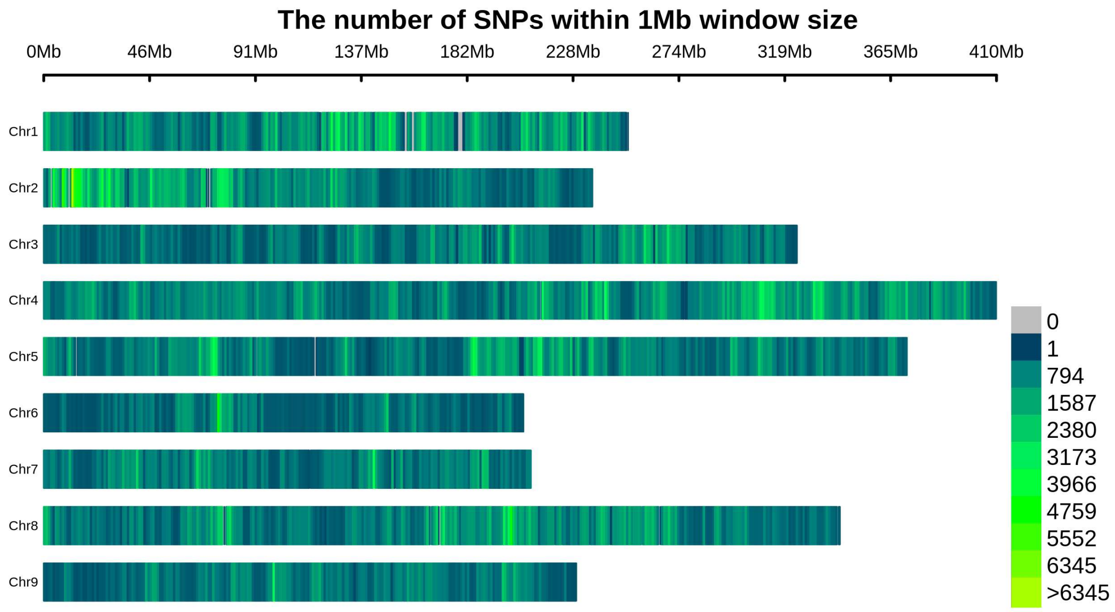

Fig. S4 ***SNP-based PCA plot of 132 L. sativa accessions used for the primary GWAS showing the first (30.5% variance explained) and second (15.2% variance explained) principal components (PCs)****. Each point represents an accession, with colours corresponding to crop types: Butterhead (green), Crisp (gold), Latin (wine), Cos (beige), Cutting (pink), Stalk (dark green), Oilseed (bright yellow). The clustering pattern highlights genetic differentiation and structure among the crop types, with much of the variation effectively captured by the first two principal components.*

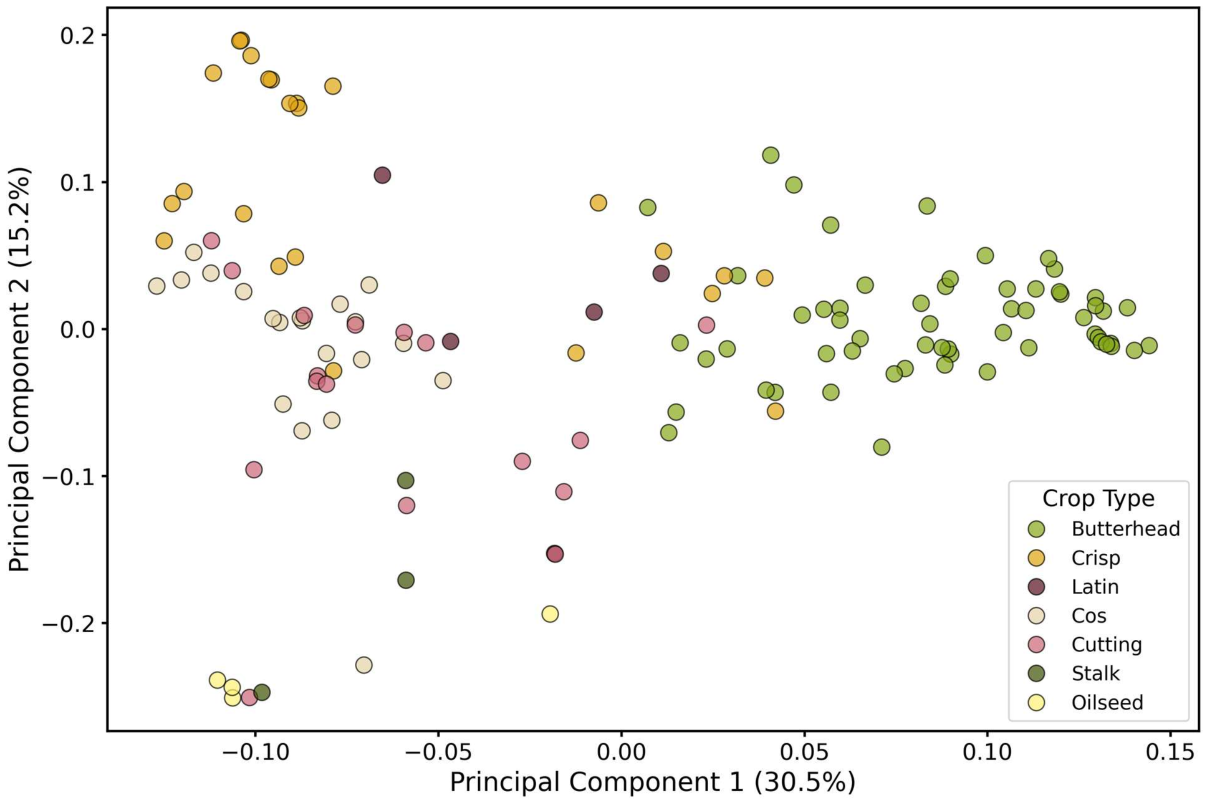

Fig. S5 ***Further examination of the cultivated lettuce extended circadian period. a.*** *Polar plot displaying phase and period data. Angles represent phase (in radians, counterclockwise direction), and the radial distance from the centre corresponds to the circadian period (in hours). Individual points represent data per accession (N ≥ 3), while outlined circles indicate the species-specific mean phase and period, color-coded per species. Mean circadian phases were compared using the Watson-Wheeler test and confirm statistical differences between L. sativa (olive) and its wild relatives (L. saligna, rose, p-value <0.0001 and L. serriola, gold, p-value=0.0002). No differences were found between L. saligna and L serriola (p-value =0.4238).* ***b.*** *Relative amplitude error (RAE) boxplot. RAE values closer to 0 indicate robust rhythmic cycling, while values closer to 1 suggest weaker or less consistent rhythms. Lower RAE values also reflect better performance of the rhythm estimation algorithms. Boxplots show median (central line), interquartile range (box), whiskers extending to data range, with accession data points (N ≥ 3), overlaid as a stripplot (dots). Significant differences are indicated: p<0.01 (**), p<0.001 (***), and non-significant (ns), based on ANOVA with Tukey HSD post-hoc tests. L. sativa (green) shows significantly lower RAE compared to L. serriola (yellow, p<0.001) and L. saligna (rose, p<0.01). No significant differences are observed between L. serriola and L. saligna.*

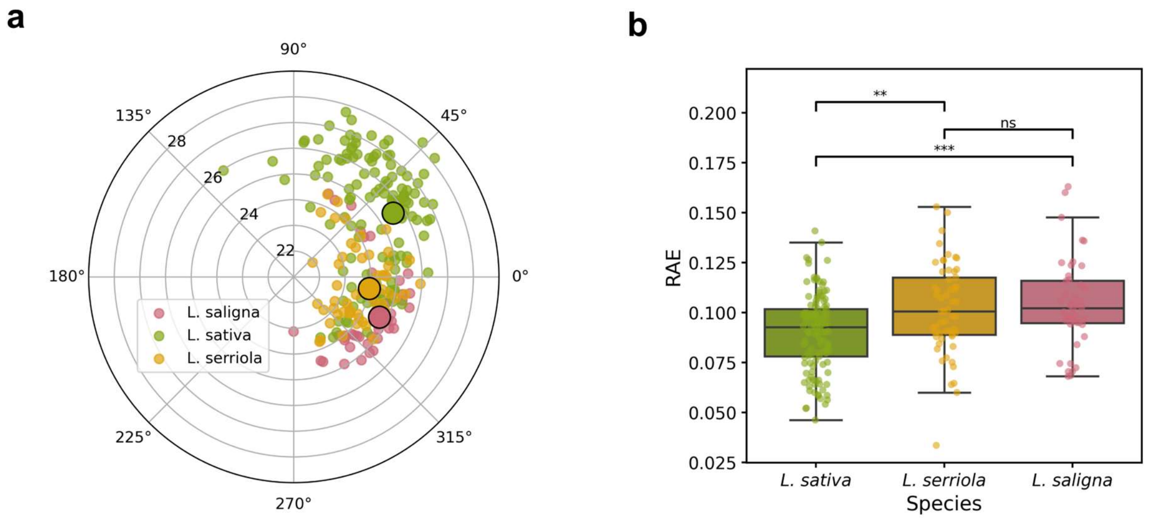
a
90°
135°
45°
28
26
24
22
180°
0°
L. saligna
L. sativa
L. serriola
225°
315°
270°
b
**
ns
***
0.200
0.175
0.150
0.125
0.100
0.075
0.050
0.025
RAE
L. sativa
L. serriola
L. saligna
Species

Fig. S6 ***Genome-wide Association Analysis (GWAS) of developmental traits in Lactuca sativa (genome v.11). a, b.*** *Manhattan and quantile-quantile (Q-Q) plots for GWAS results from the screening of inflorescence emergence time (days until 55) and flowering time (days until 60). The strongest association is observed on chromosome 7 (166–168 Mb), where our candidate gene PHYTOCHROME C (PHYC) resides.* ***c.*** *Manhattan plot for flowering time using publicly available data from the CGN dataset (Wei et al., 2021), confirming the strong signal at the PHYC locus on chromosome 7 previously reported (v8 genome assembly).* ***d-g****. Manhattan and Q-Q plots for developmental stages obtained through destructive phenotyping: start to bolt (**d**), bolting time (**e**), start to bud (**f**), and budding time (g). The PHYC locus consistently emerges as the strongest signal for bolting time and budding time, highlighting its critical role in these processes. The dashed horizontal grey line represents the Bonferroni-corrected significance threshold ($\alpha = 0.05$, $2.29 \cdot 10^{-8}$), and the genomic inflation factor (λGC) is indicated in each Q-Q plot.*

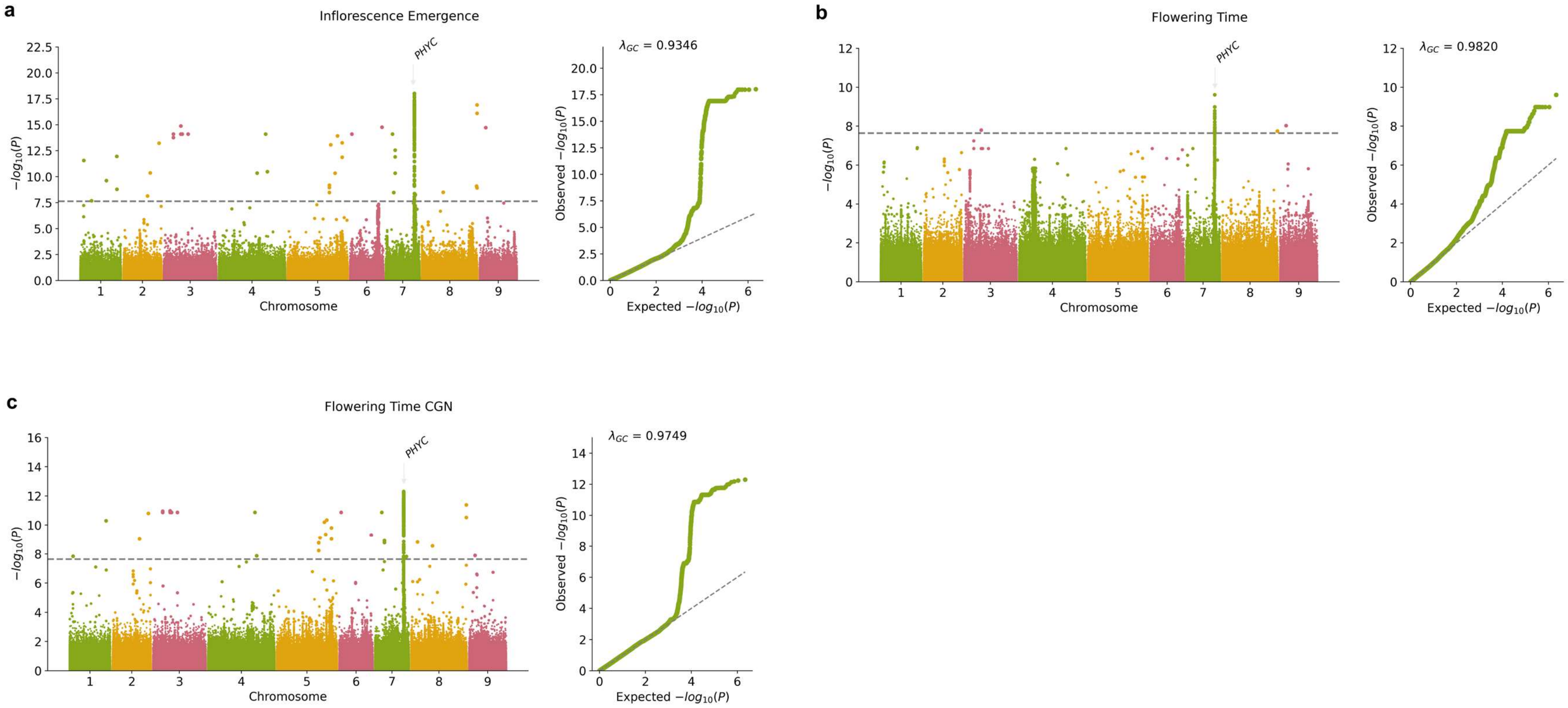
a
Inflorescence Emergence
PHYC
−log10(P)
Chromosome
λGC = 0.9346
Observed −log10(P)
Expected −log10(P)
b
Flowering Time
PHYC
−log10(P)
Chromosome
λGC = 0.9820
Observed −log10(P)
Expected −log10(P)
c
Flowering Time CGN
PHYC
−log10(P)
Chromosome
λGC = 0.9749
Observed −log10(P)
Expected −log10(P)

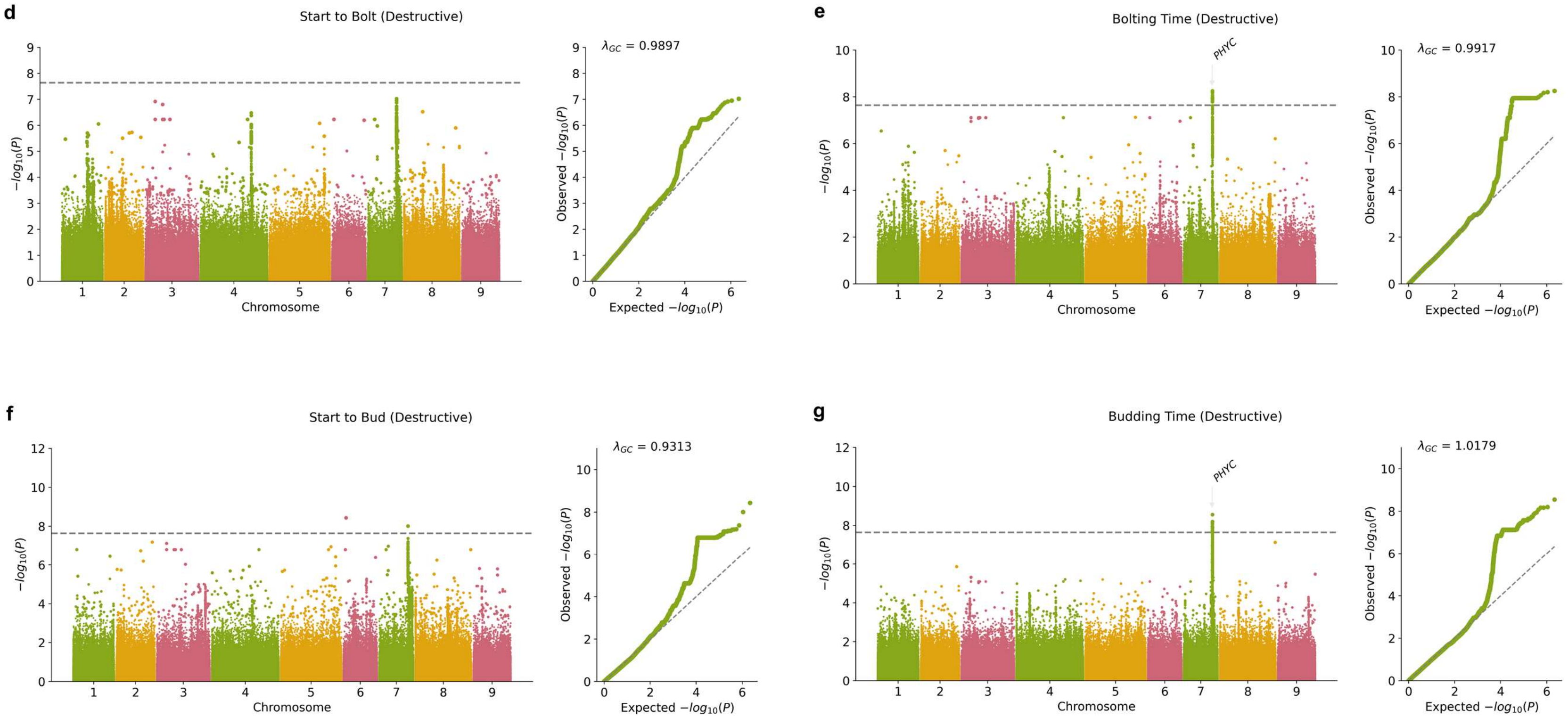
d
Start to Bolt (Destructive)
−log10(P)
Chromosome
λGC = 0.9897
Observed −log10(P)
Expected −log10(P)
e
Bolting Time (Destructive)
PHYC
−log10(P)
Chromosome
λGC = 0.9917
Observed −log10(P)
Expected −log10(P)
f
Start to Bud (Destructive)
−log10(P)
Chromosome
λGC = 0.9313
Observed −log10(P)
Expected −log10(P)
g
Budding Time (Destructive)
PHYC
−log10(P)
Chromosome
λGC = 1.0179
Observed −log10(P)
Expected −log10(P)

Fig. S7 ***Regional manhattan plot of GWAS results for circadian clock period.*** *Zoom into Chromosome 7: 175-178 Mb. The horizontal dashed line represents the Bonferroni-corrected threshold for genome-wide significance (α = 0.05). The most significant SNP (Chr7:176812430) is located within the PHYC locus (176809300-176814445) with a p-value of* 4.28×10^{-14}*, causing a frame-shift mutation. The red dot represents this SNP and the dashed red vertical line its position.*

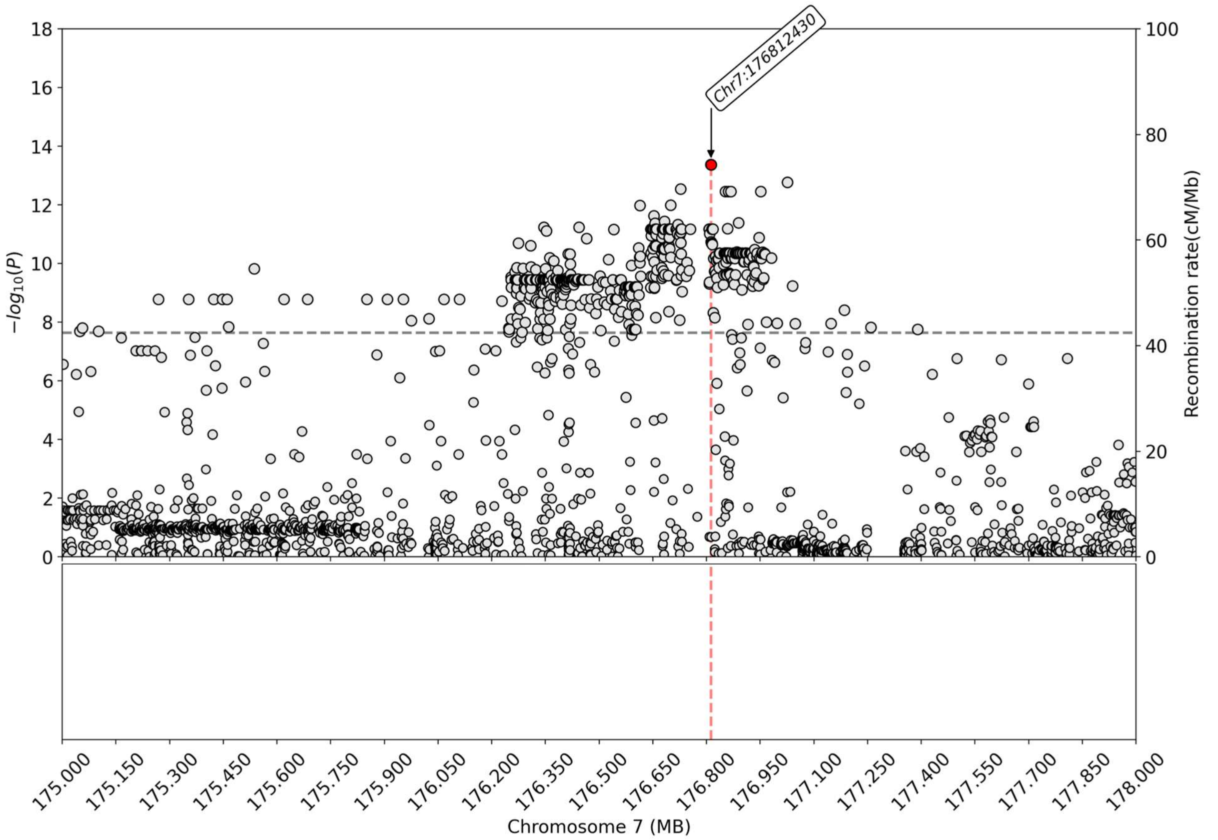

Fig. S8 ***Genome-wide association analysis (GWAS) of developmental traits and circadian clock in Lactuca sativa (genome v.11).*** *Multitrack Manhattan and quantile-quantile (Q-Q) plots display GWAS results, color-coded by the algorithm used (blue: GLM, yellow: MLM, purple: FarmCPU).* ***a-b.*** *Circadian clock period and bolting time GWAS results, highlighting a strong signal at Chr7:168 Mb, where the PHYC locus resides, consistent with findings using our main algorithm (EMMAX).* ***c-d.*** *Visually scored inflorescence emergence and flowering time, showing the strongest association again in the PHYC locus****. e-h.*** *Destructively phenotyped traits, including start of bolting (e), bolting (f), start of budding (g), and budding (h), revealing the same significant signal when detected. i. Flowering time GWAS results from publicly available data, aligning with our EMMAX-based results and previous research. The dashed red line represents the Bonferroni corrected significance threshold ($\alpha = 0.05$, $2.29 \cdot 10^{-8}$). The Manhattan plots highlight significant associations across chromosomes, with many strong signals at Chr7:168 Mb.*

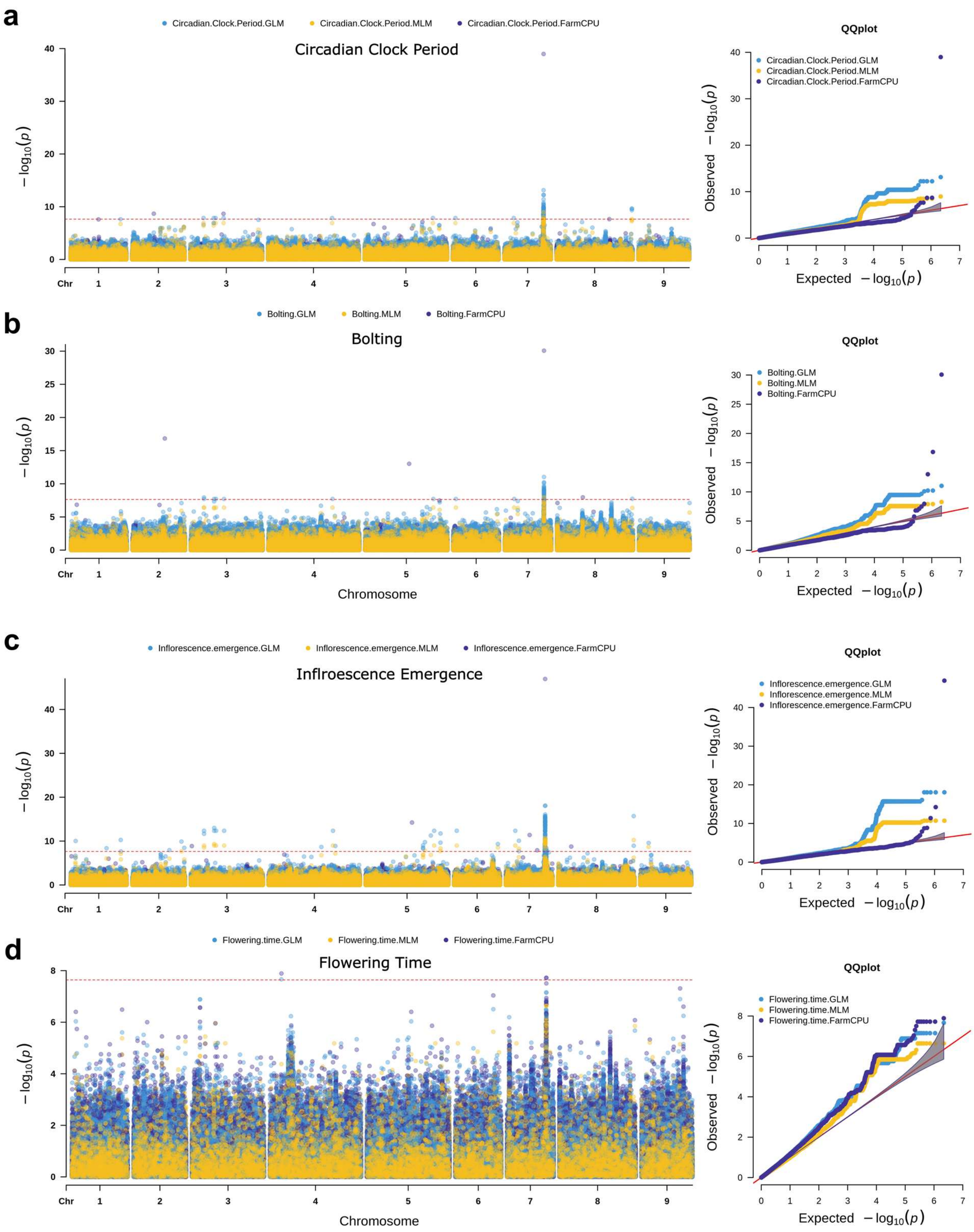
a
Circadian.Clock.Period.GLM
Circadian.Clock.Period.MLM
Circadian.Clock.Period.FarmCPU
Circadian Clock Period
QQplot
b
Bolting.GLM
Bolting.MLM
Bolting.FarmCPU
Bolting
Chromosome
c
Inflorescence.emergence.GLM
Inflorescence.emergence.MLM
Inflorescence.emergence.FarmCPU
Infloescence Emergence
d
Flowering.time.GLM
Flowering.time.MLM
Flowering.time.FarmCPU
Flowering Time
$-\log_{10}(p)$
Chr
Observed $-\log_{10}(p)$
Expected $-\log_{10}(p)$

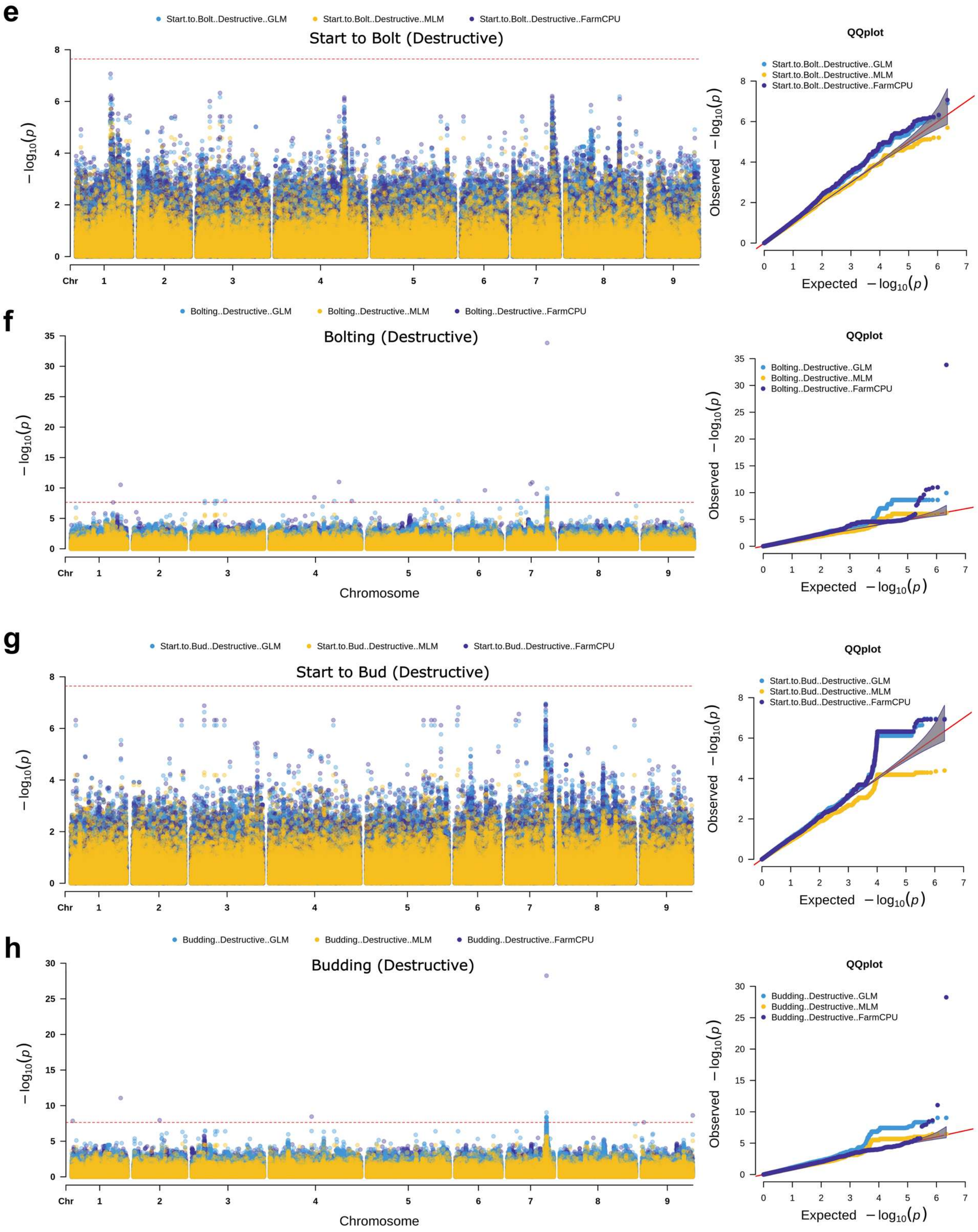
e
Start.to.Bolt..Destructive..GLM
Start.to.Bolt..Destructive..MLM
Start.to.Bolt..Destructive..FarmCPU
Start to Bolt (Destructive)
QQplot
Chr 1 2 3 4 5 6 7 8 9
f
Bolting..Destructive..GLM
Bolting..Destructive..MLM
Bolting..Destructive..FarmCPU
Bolting (Destructive)
QQplot
Chromosome
g
Start.to.Bud..Destructive..GLM
Start.to.Bud..Destructive..MLM
Start.to.Bud..Destructive..FarmCPU
Start to Bud (Destructive)
QQplot
h
Budding..Destructive..GLM
Budding..Destructive..MLM
Budding..Destructive..FarmCPU
Budding (Destructive)
QQplot
Chromosome
Observed $-\log_{10}(p)$
Expected $-\log_{10}(p)$
$-\log_{10}(p)$

i

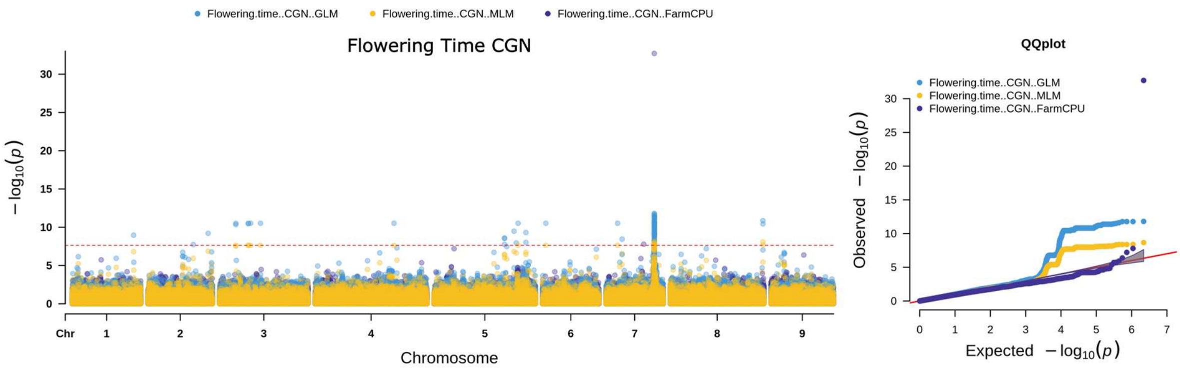

Flowering.time..CGN..GLM
Flowering.time..CGN..MLM
Flowering.time..CGN..FarmCPU
Flowering Time CGN
$-\log_{10}(p)$
30
25
20
15
10
5
0
Chr
1
2
3
4
5
6
7
8
9
Chromosome
QQplot
Flowering.time..CGN..GLM
Flowering.time..CGN..MLM
Flowering.time..CGN..FarmCPU
Observed $-\log_{10}(p)$
Expected $-\log_{10}(p)$
0
1
2
3
4
5
6
7

Fig. S9 ***Genome-wide association analysis (GWAS) of circadian clock and developmental traits in Lactuca sativa (genome v.11) with key SNP in the PHYC locus (Chr7:176812430) as known effect.*** *Manhattan and quantile-quantile (Q-Q) plots for GWAS results from our phenotyping data. No significant signals were detected when including PHYC as a covariate in our analysis. From top left to bottom: Start to bolting (destructive), Bolting time (destructive), Start to Budding (destructive), Budding (destructive), Circadian Clock Period, Bolting, Inflorescence Emergence and Flowering Time, plus Flowering Time from the CGN publicly available data. The dashed horizontal grey line represents the Bonferroni-corrected significance threshold (α = 0.05, $2.29 \cdot 10^{-8}$), and the genomic inflation factor (λGC) is indicated in each Q-Q plot.*

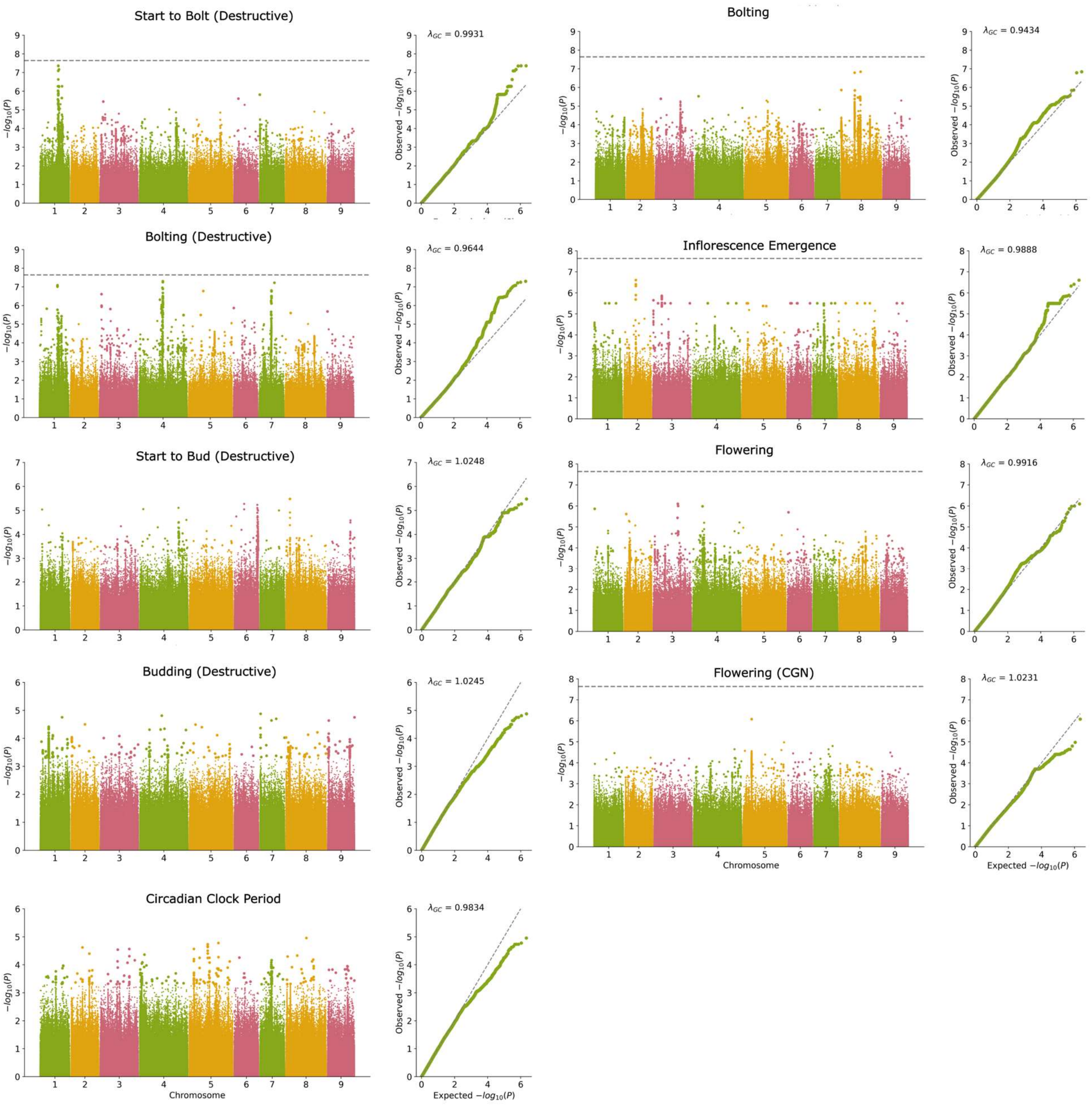
Start to Bolt (Destructive)
λGC = 0.9931
Bolting
λGC = 0.9434
Bolting (Destructive)
λGC = 0.9644
Inflorescence Emergence
λGC = 0.9888
Start to Bud (Destructive)
λGC = 1.0248
Flowering
λGC = 0.9916
Budding (Destructive)
λGC = 1.0245
Flowering (CGN)
λGC = 1.0231
Circadian Clock Period
λGC = 0.9834
−log10(P)
Observed −log10(P)
Expected −log10(P)
Chromosome

Fig. S10 ***Validation of PHYC variant distribution across Lactuca species and haplotype assignments.*** *Stacked bar plot of predominant variants detected in our haplotyping analysis, showing their prevalence and allele counts for each Lactuca species. Variants were assigned to haplotypes (represented by coloured dots) through SNP phasing in the PHYC region and subsequently grouped by similarity to form haplogroups.*

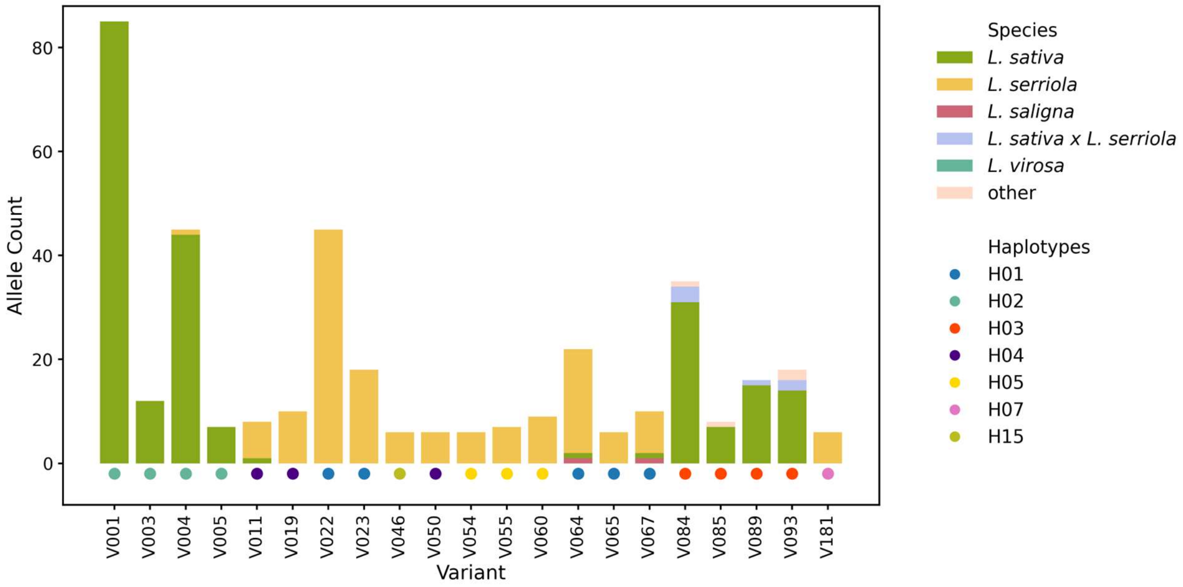

Fig. S11 ***Receiver operating characteristic (ROC) analysis illustrating discriminative power of the PHYC lettuce H02 haplotype.*** *ROC curves showing the ability of PHYC H02 haplotype presence to predict circadian clock period deceleration, delayed bolting time (non-destructive, days until 51), and delayed flowering time (CGN) in cultivated lettuce. Each curve represents the trade-off between true positive rate (sensitivity) and false positive rate (1-specificity) across all possible classification thresholds. The area under the curve (AUC) values is indicated in each plot: 0.968, 0.910, and 0.911 for circadian period, bolting time, and flowering time, respectively, all exceed the threshold of 0.9 considered excellent discrimination. The diagonal dashed line represents random chance (AUC = 0.5). These results demonstrate that H02 functions as a highly effective binary predictor of both circadian (~97% accuracy) and developmental phenotypes (~91& accuracy), providing quantitative support for its role as a major-effect locus controlling clock-development integration in lettuce.*

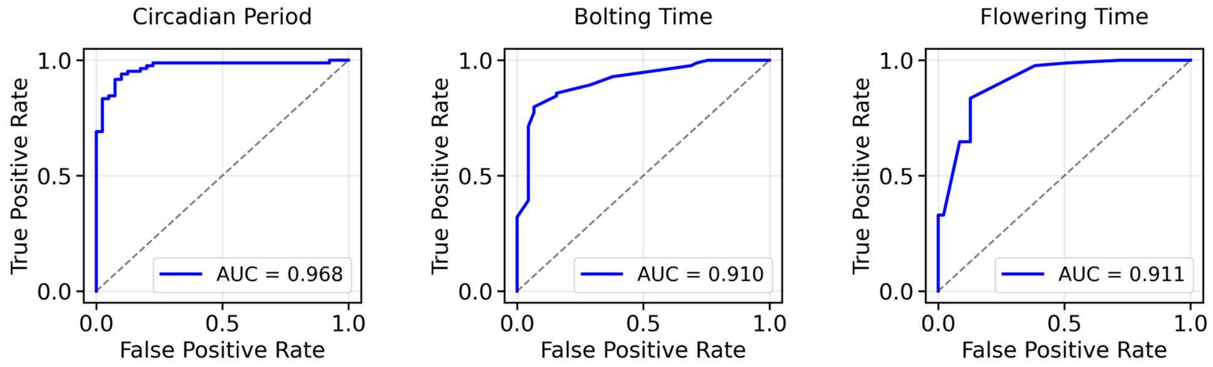

Supplement: Supplementary file 1 — Fig. S1 Drone‐captured top view of the experimental field used for scoring developmental traits. Fig. S2 Chronological representation of four bolting stages scored through destructive measurements. Fig. S3 Distribution of SNPs after hard filtering and LD pruning. Fig. S4 SNP‐based PCA plot of 132 Lactuca sativa accessions used for the primary GWAS showing the first (30.5% variance explained) and second (15.2% variance explained) principal components (PCs). Fig. S5 Further examination of the cultivated lettuce extended circadian period. Fig. S6 Genome‐wide Association Analysis (GWAS) of developmental traits in Lactuca sativa (genome v.11). Fig. S7 Regional Manhattan plot of GWAS results for circadian clock period. Fig. S8 Genome‐wide association analysis (GWAS) of developmental traits and circadian clock in Lactuca sativa (genome v.11). Fig. S9 Genome‐wide association analysis (GWAS) of circadian clock and developmental traits in Lactuca sativa (genome v.11) with key SNP in the PHYC locus (Chr7:176812430) as known effect. Fig. S10 Validation of PHYC variant distribution across Lactuca species and haplotype assignments. Fig. S11 Receiver operating characteristic (ROC) analysis illustrating the discriminative power of the PHYC lettuce H02 haplotype. [file NPH-248-1892-s001.pdf]
